# Supplementary material for: Emergence of global scaling behaviour in the coupled Earth-atmosphere interaction
Source: Sci Rep. 2016 Sep 26;6:34005. doi: 10.1038/srep34005 (PMC5036056; doi:10.1038/srep34005)
Supplement: Supplementary Information [file srep34005-s1.pdf]

Supplementary Information for "Emergence of global scaling behaviour in the coupled Earth-atmosphere interaction"

Bijan Fallah, Abbas Ali Saber and Sahar Sodoudi

January 5, 2016

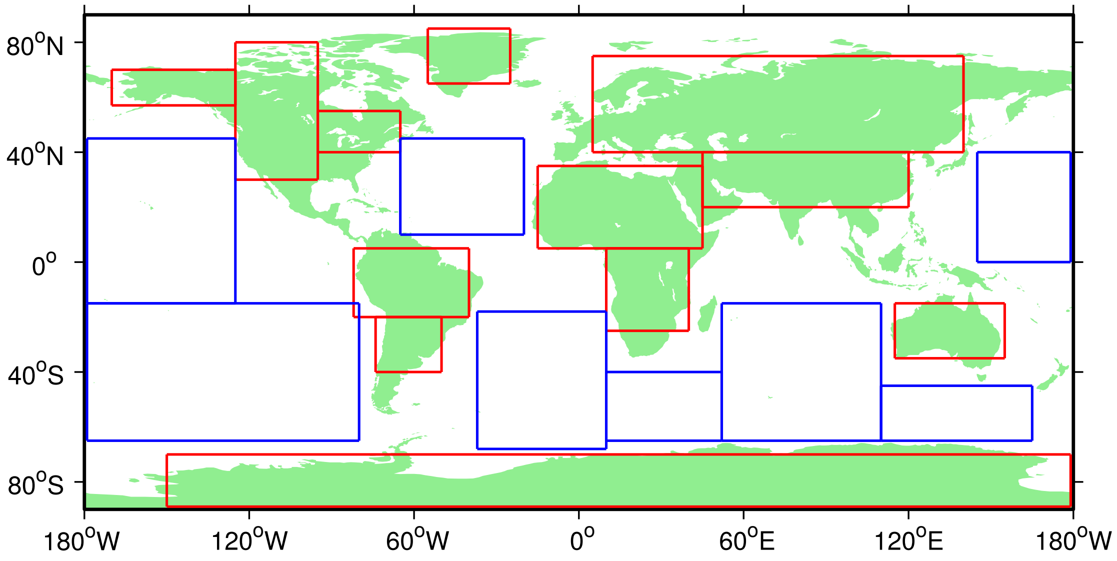

Figure 1: Schematic showing the land (red boxes) and ocean (blue boxes) regions selected for the analysis.This figure is created by Mapping Toolbox of MATLAB (`http://www.mathworks.com/`) version R2010b.

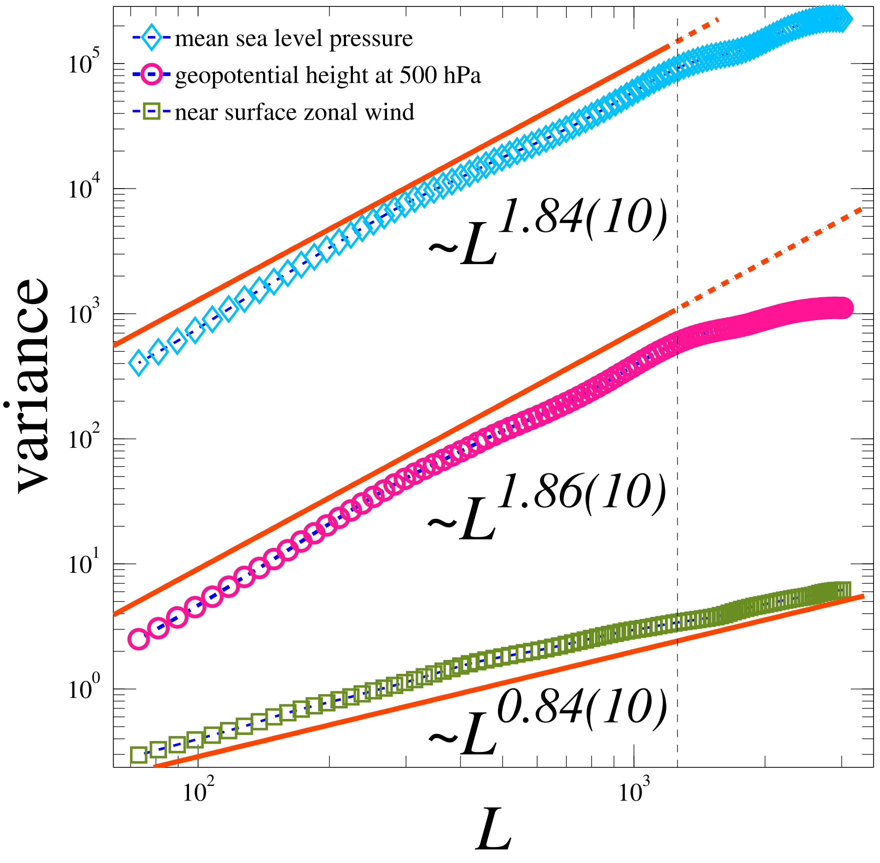

Figure 2: The plot of variance of near surface zonal wind (squares), geopotential height at 500 hPa (circles) and mean sea level pressure (diamonds) within the boxes that are randomly chosen. Solid line indicates the power-law fit.
